# Effects of Graphene Oxide on Endophytic Bacteria Population Characteristics in Plants from Soils Contaminated by Polycyclic Aromatic Hydrocarbons

**DOI:** 10.3390/molecules29102342

**Published:** 2024-05-16

**Authors:** Xingxing Zhou, Bo Zhang, Qingzhu Meng, Lingmei Li

**Affiliations:** 1 College of Architecture and Environment, Ningxia Institute of Science and Technology, Shizuishan 753000, China; 2010419@stu.neu.edu.cn; 2 Key Laboratory of Ministry of Education on Safe Mining of Deep Metal Mines, Northeastern University, Shenyang 110819, China; 2010420@stu.neu.edu.cn; 3 College of Material Science and Green Technologies, Kazakh-British Technical University, Almaty 050000, Kazakhstan; q_meng@kbtu.kz; 4 College of Life Science, Shenyang Normal University, Shenyang 110034, China

**Keywords:** phytoremediation, PAH-contaminated soil, graphene oxide, endophytic bacteria, microbial community analysis

## Abstract

Environmental pollution stands as one of the significant global challenges we face today. Polycyclic aromatic hydrocarbons (PAHs), a class of stubborn organic pollutants, have long been a focal point of bioremediation research. This study aims to explore the impact and mechanisms of graphene oxide (GO) on the phytoremediation effectiveness of PAHs. The results underscore the significant efficacy of GO in accelerating the degradation of PAHs. Additionally, the introduction of GO altered the diversity and community structure of endophytic bacteria within the roots, particularly those genera with potential for PAH degradation. Through LEfSe analysis and correlation studies, we identified specific symbiotic bacteria, such as *Mycobacterium*, *Microbacterium*, *Flavobacterium*, *Sphingomonas*, *Devosia*, *Bacillus*, and *Streptomyces*, which coexist and interact under the influence of GO, synergistically degrading PAHs. These bacteria may serve as key biological markers in the PAH degradation process. These findings provide new theoretical and practical foundations for the application of nanomaterials in plant-based remediation of polluted soils and showcase the immense potential of plant–microbe interactions in environmental restoration.

## 1. Introduction

Polycyclic aromatic hydrocarbons (PAHs) represent a group of organic molecules characterized by the presence of multiple interconnected benzene rings, widely distributed in soil, water, and air [1]. PAHs have high stability, low water solubility, high lipophilicity and bioaccumulation, and can enter the soil through atmospheric deposition, surface runoff, subsurface leaching, and other pathways, causing long-term soil pollution [2,3]. PAHs have potential toxicity and carcinogenicity to ecosystems and human health and have been classified as class I or II carcinogens by the World Health Organization (WHO) and the International Agency for Research on Cancer (IARC) [4,5,6,7]. Therefore, the remediation and management of PAH-contaminated soil is an urgent problem to be solved.

At present, physical, chemical, and biological remediation are the main methods for treating PAH-contaminated soil [8]. Among these, biological remediation technology has attracted widespread attention and application due to its advantages: low cost, simple operation, and no secondary pollution [9]. Bioremediation is a process that employs the interaction between plants and microorganisms to extract both organic and inorganic pollutants from an affected setting [10]. This method utilizes the natural breakdown abilities of soil-dwelling microbes to diminish, eradicate, gather, stabilize, and expedite the decomposition of contaminants within the soil [11]. Phytoremediation technology mainly relies on microorganisms or plants and their endophytic microorganisms to degrade or transform PAHs, thus reducing or eliminating the toxicity of PAHs to the environment and organisms. Recently, several authors have demonstrated that endophytic bacteria have distinct degrading potentials for PAHs [12,13]. However, due to factors such as the structural stability, low bioavailability, and biodegradation difficulty of PAHs, the efficiency and effectiveness of biological remediation technology still need to be improved [14,15].

In recent years, nanomaterials, as a new type of bioremediation enhancer, have attracted the interest and attention of researchers. Nanomaterials have the characteristics of a high specific surface area, high reactivity, high adsorption capacity, and high catalytic efficiency, which can affect or improve the bioremediation process in a variety of ways, such as improving the bioavailability of pollutants and enhancing the degradation ability of microorganisms [16,17]. Contemporary research indicates that the incorporation of nanomaterials into soil can enhance the phytoremediation efficiency for organic substances [17]. Nano-scale zero-valent iron particles have been found to break down organic contaminants, including atrazine and chlorpyrifos. Additionally, nanoparticles can play a role in enzymatic bioremediation methods [18].

Graphene oxide (GO) is a derivative of graphene that has various oxygen-containing functional groups on its surface [19]. Due to its unique physicochemical properties and structure, GO has significant applications in the fields of medicine, environmental science, and material research [20]. For instance, GO can be used to synthesize a novel adsorbent (MS@RGO) that has demonstrated remarkable performance in removing marine pollution [21]; GO can also effectively treat organic pollutants in wastewater [22]. Moreover, the oxygen atoms on GO can form complexes with metal ions, which endows GO with the ability to adsorb different kinds of heavy metal ions and play a vital role in the removal of heavy metals [23]. These studies suggest that GO is a bright prospect in the remediation of various pollutants. However, few studies have explored the potential of GO in enhancing plant accumulation and degradation of PAHs. Therefore, it is imperative to investigate the role of GO in bioremediation.

Endophytic bacteria are bacteria that parasitize plants and form a mutually beneficial symbiotic relationship with them. Endophytic bacteria can provide nutrients, hormones, antibiotics, etc. that plants need to enhance their growth and resistance. Endophytic bacteria can also participate in the degradation of pollutants, enhancing the tolerance and removal ability of plants to pollutants [8]. Endophytic bacteria have been identified as crucial agents in the breakdown of pollutants within contaminated sites. Specific endophytic microbes, extracted from a variety of plants, exhibit a greater capacity for organic pollutant decomposition than native soil bacteria [24,25]. It is well established that these bacteria not only significantly boost plant biomass production but also enhance petroleum degradation more effectively than bacteria limited to hydrocarbon degradation [26,27]. Furthermore, endophytic bacteria can generate diverse hydrolytic enzymes implicated in the disintegration of plant materials, which often share chemical similarities with toxic organic pollutants like petroleum hydrocarbons. In the study by Gutiérrez-Ginés et al., *Lupinus luteus* was utilized for the remediation of soils tainted with organic contaminants, including benzo[a]pyrene and various PAHs, and the findings revealed that endophytic bacteria can enhance plant growth and increase resilience to benzo[a]pyrene and other organic pollutants such as diesel fuel and polychlorinated biphenyls (PCBs). Moreover, certain bacterial strains demonstrated the capability to metabolize these organic pollutants [28]. Consequently, endophytic bacteria possess significant biochemical potential and display an innate proficiency in the degradation of xenobiotics during phytoremediation processes [29].

This research aimed to explore the influence and underlying processes of GO on the phytoremediation of PAHs. This study assessed the effects of GO on the composition and functionality of endophytic bacterial communities. Utilizing Fire Phoenix (*Festuca* spp.), known for its robust PAH degradation capabilities, varying levels of GO (0 and 0.4 g·kg^−1^) were integrated into the soil. The phytoremediation efficacy and the operational dynamics of GO on PAHs were determined by quantifying the PAH concentrations in plant tissues and by high-throughput sequencing to analyze the endophytic bacterial community structure. The findings offer fresh theoretical and practical insights for employing GO in phytoremediation and provide a novel perspective and methodology to further clarify the impact and workings of GO on endophytic bacteria.

## 2. Results and Discussion

### 2.1. GO Promoted PAHs’ Degradation and Absorption

**Figure 1:**
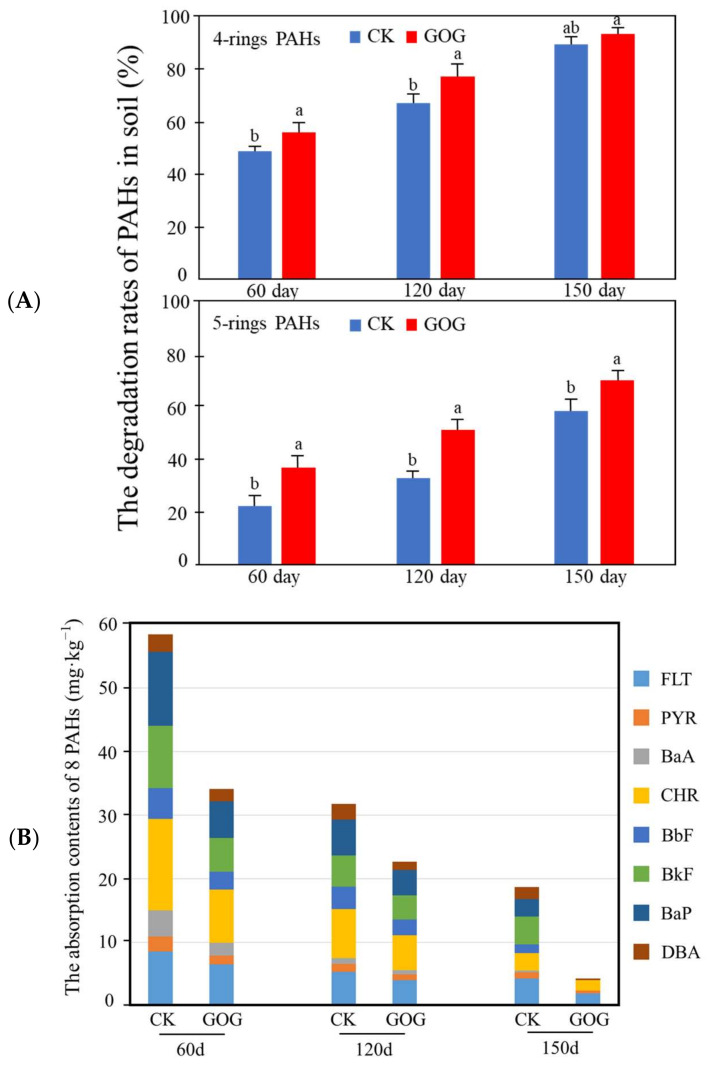
The degradation rates of PAHs in soil and the content of PAHs in plants. (**A**) The degradation rates of PAHs in soil under different concentrations of GO (0 and 0.4 g·kg^−1^) and different timeframes (60, 120, and 150 d); The a and b on the bar graphs indicate significant differences between CK and GOG, with a, b labeled from largest to smallest. (**B**) the content of PAHs in plants under different concentrations of GO (0 and 0.4 g·kg^−1^) and different timeframes (60, 120, and 150 d).

Figure 1A illustrates the impact of GO on the degradation rates of four-ring PAHs (FLT, PYR, BaA, CHR) and five-ring PAHs (BbF, BkF, BaP, DBA). The detection results of GC-MS revealed that the degradation rates of Σ8 PAHs in the graphene oxide group (GOG) were 44.40%, 61.99%, and 78.95% at 60, 120, and 150 d, respectively, which were 9.72%, 13.45%, and 6.78% higher than those of the CK at the corresponding time points, indicating that GO significantly enhanced the degradation of PAHs in the rhizosphere soil. A generally higher degradation rate of four-ring PAHs than five-ring PAHs was observed throughout the phytoremediation cycle. However, the GOG was more efficient in promoting the degradation of five-ring PAHs than four-ring PAHs. At 60 days, the degradation rate of four-ring Σ4 PAHs in the GOG was 58.63%, which was 5.77% higher than the CK, whereas the degradation rate for five-ring Σ4 PAHs was 33.74–12.61% higher than the CK. At 120 days, the degradation rate for four-ring Σ4 PAHs in the GOG increased to 80.63%, which was 10.04% higher than that of the CK, while the degradation rate of five-ring Σ4 PAHs was 44.21–13.93% higher than the CK. By 150 days, the degradation rate for four-ring Σ4 PAHs in the GOG reached 94.10%, 4.28% higher than the CK, while the degradation rate for five-ring Σ4 PAHs was 67.84%, which was 11.42% higher than that of the CK. This result suggests that GO can effectively improve the degradation of higher-ring PAHs. As observed in Figure 1B, in the natural state (CK), the accumulated content of Σ8 PAHs in Fire Phoenix were 58.47 mg·kg^−1^, 31.76 mg·kg^−1^, and 18.49 mg·kg^−1^ at 60, 120, and 150 days, respectively. Following the addition of GO, the accumulation decreased to 34.17 mg·kg^−1^, 22.73 mg·kg^−1^, and 4.32 mg·kg^−1^, respectively. Notably, after 150 days, five-ring PAHs such as BbF, BkF, and BaP were undetectable, indicating that GO significantly enhanced the degradation of PAHs in plants, particularly those with higher molecular weight PAHs.

In recent years, eco-friendly nanomaterials have emerged as a focal point among researchers and scholars worldwide in the field of organic soil contamination remediation. Numerous studies have demonstrated that nanomaterials can directly or indirectly engage in the breakdown of organic pollutants, boasting a level of remediation efficiency that surpasses that of conventional materials [30]. Certain nanomaterials have demonstrated significant potential in the degradation of organic pollutants. Notably, zero-valent nano-iron leverages its robust adsorption capabilities and redox properties to degrade up to 90% of chlorpyrifos in soil within a mere 10 days [31]. Additionally, it markedly improves the degradation efficiency of pyrene in soil [32]. Low concentrations of fullerenes can enhance the mobility of PCBs and phenanthrene in soil, thereby facilitating their degradation [33]. Our study indicated that GO facilitated the degradation of PAHs. Similar results have been consistently observed in multiple studies. Torre-Roche et al. have elucidated the impact of nanomaterials on the operation and uptake of pesticides and organic pollutants within plants [34]. The application of carbon-based nanomaterials has notably increased plant uptake of DDE (a metabolite of DDT) from soil, evidenced by a 29% increase in zucchini’s maximum absorption capacity for DDE [35]. This enhancement is likely due to the diffusion of PAHs to the plant root surface, where they traverse into the plant through both symplastic and apoplastic routes, subsequently accumulating via lignification [36]. Once internalized, PAHs undergo metabolic or mineralization transformations into carbon dioxide and water, or into non-toxic intermediate metabolites stored within the plant cells [37]. Fang et al. suggested that the diminutive size of nanomaterials allows them to penetrate the plant’s root system via xylem and phloem, facilitating nutrient transport and bolstering the plant’s pollutant absorption capacity, thereby increasing pollutant removal [38]. These insights are in alignment with the outcomes of our study. Considering the sub^−10^ nm size of GO utilized herein, it is plausible that plants can directly absorb these nanomaterials, thereby bolstering the phytoremediation potential against PAH-contaminated soil. Consequently, the intrinsic plant degradation pathways represent a significant mechanism within the remediation framework and warrant considerable recognition.

### 2.2. GO Increased Endophytic Bacterial Diversity

Root samples gathered at various stages of phytoremediation (60, 120, and 150 d) underwent analysis for endophytic bacterial populations via high-throughput sequencing. Alpha diversity analysis, which assesses the variety and abundance of microbial communities, served as a reflection of the environmental microorganism diversity. Post-sequencing (Table 1), the diversity and abundance of the endophytic bacterial communities in the plant roots were determined with a 97% similarity threshold. The Shannon and Simpson indices are metrics that gauge the diversity of the endophytic bacterial community within the roots. A higher Shannon index and a lower Simpson index denote a more diverse community. Throughout all three timeframes, the Shannon index was elevated for GO compared to the CK, while the Simpson index was higher for the CK, suggesting that GO’s presence bolstered the endophytic bacterial diversity in the roots. The Ace and Chao indices, indicative of community richness, were also higher for GO, implying that GO’s inclusion boosted the endophytic bacterial population. Furthermore, a coverage estimate exceeding 99% confirmed the adequacy of the high-throughput sequencing method in capturing the dynamics of the roots’ endophytic microbial community.

### 2.3. GO Changed the Endophytic Microbial Community Structure

**Figure 2:**
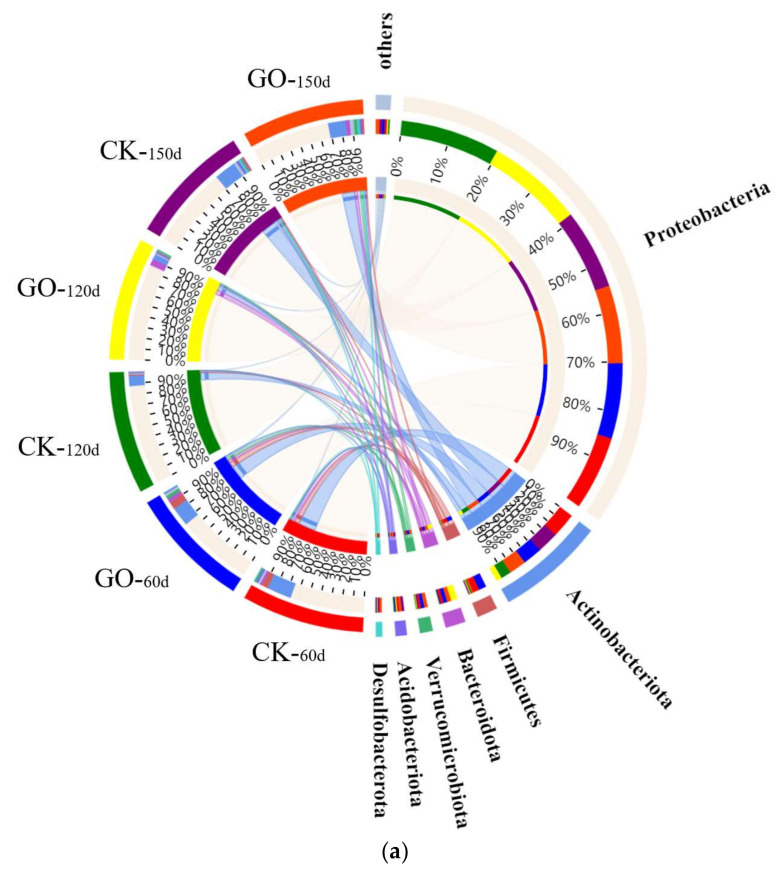

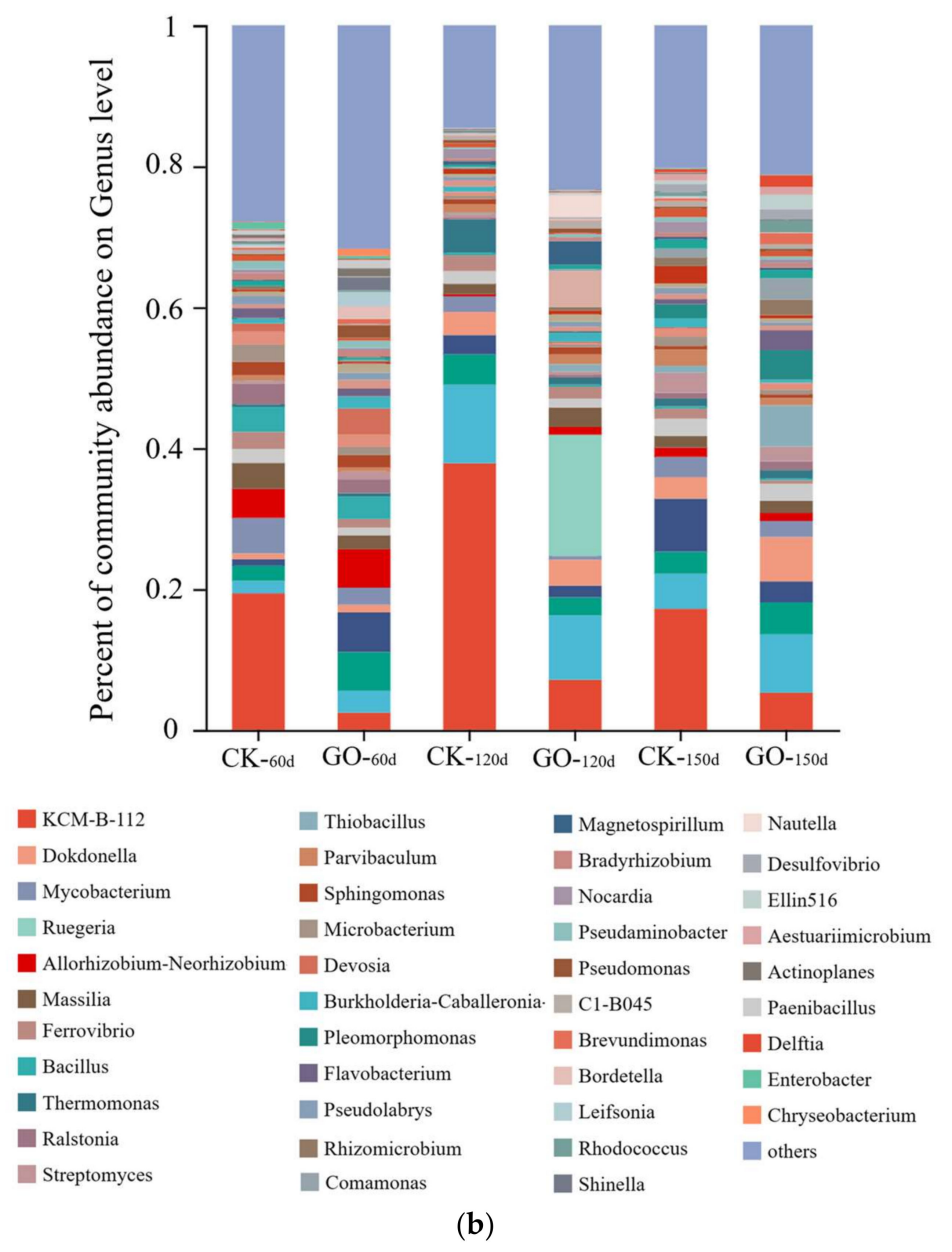
Effect of GO on the endophytic microbial community structure. (**a**) Distribution of phylum in the endophytic bacteria in different groups based on the taxonomy annotation from NCBI-NR database in 60 d, 120 d, and 150 d, respectively. The width of each ribbon indicates the abundance of each taxonomic group. The solid tick marks above the inner circle denote the actual read counts, while the tick marks above the outer circle represent the proportional abundance of each taxon. (**b**) Changes in the composition of the dominant species in different samples (CK and GO) at genus level under different timeframes (60, 120, and 150 d).

Endophytic bacterial OTUs obtained by Illumina 16S rRNA gene sequencing were categorized into 28 distinct phyla. Figure 2a illustrated the distribution of endophytic bacterial phyla. Sequencing revealed that the nine predominant endophytic bacterial phyla are *Proteobacteria*, *Actinobacteriota*, *Firmicutes*, *Bacteroidota*, *Verrucomicrobiota*, *Acidobacteriota*, *Desulfobacterota*, *Myxococcota*, and *Bdellovibrionota*. These phyla accounted for 97.55% to 99.64% of the overall endophytic bacterial abundance. Notably, *Proteobacteria*, *Actinobacteriota*, *Firmicutes*, and *Bacteroidota* emerged as the leading phyla within the samples. The addition of GO resulted in an increased relative abundance of *Proteobacteria* and *Bacteroidota*, particularly notable at 120 d. *Actinobacteria*, the second most dominant phylum among endophytic bacteria, exhibited a decrease in relative abundance due to GO supplementation, with reductions of 4.81%, 4.32%, and 3.84% at 60 d, 120 d, and 150 d, respectively, compared to the control (CK). Similarly, the relative abundance of *Firmicutes* was diminished following GO treatment. These dominant phyla have also been identified as the primary phyla in other soils contaminated with PAHs, underscoring their significant potential for PAH biodegradation [39,40]. The *Proteobacteria* phylum is pivotal in the earth’s carbon, nitrogen, and sulfur cycles due to its metabolic versatility. Predominantly, it houses denitrifiers that can expedite soil nitrogen removal, impacting soil health and ecosystem balance [41]. They compensate for the plant’s inherent deficiencies in degrading or metabolically transforming exogenous pollutants such as PAHs by directly degrading PAHs. Furthermore, many microbes play a pivotal role in nitrogen fixation and the nitrogen cycle, effectively enhancing root absorption of nitrogenous nutrients, promoting the growth and development of roots and plants, and thereby increasing plant tolerance to pollution [40,42]. The evolution of genus-level endophytic bacterial communities upon the addition of GO is depicted in Figure 2b. The introduction of GO significantly alters the community structure of endophytic bacteria at the genus level. These dominant genera are mainly KCM-B-112, *Dokdonella*, *Massilia*, *Devosia*, *Ferrovibrio*, *Thermomonas*, *Sphingomonas*, *Ruegeria*, etc. from *Proteobacteria*; *Mycobacterium*, *Streptomyces*, *Microbacterium*, *Nocardia*, etc. from *Actinobacteriota*; *Bacillus* from *Firmicutes*; and *Flavobacterium* from *Bacteroidota*. After the addition of GO, the relative abundance of several dominant genera significantly increased. These genera include *Dokdonella*, *Ruegeria*, *Thiobacillus*, *Sphingomonas*, *Devosia*, and *Flavobacterium*. When compared to the control (CK), these genera consistently showed an upward trend in relative abundance at 60 d, 120 d, and 150 d. Specifically, at 150 d, *Dokdonella*, *Thiobacillus*, and *Flavobacterium* exhibited relative abundance increases of 3.26%, 4.86%, and 2.12%, respectively. *Ruegeria* emerged as a dominant genus in the GOG at 120 d, while *Devosia* showed a relative abundance increase of 2.58% at 60 d. The dominant genera whose relative abundance was significantly reduced by the addition of GO were KCM-B-112, *Mycobacterium*, *Ferrovibrio*, *Bacillus*, *Thermomonas*, *Streptomyces*, *Microbacterium*, *Parvibaculum*, *Nocardia*, and so on. Compared to the CK, the relative abundance of KCM-B-112 decreased by 16.93%, 30.73%, and 11.9%; *Mycobacterium* decreased by 2.26%, 1.70%, and 0.61%; and *Ferrovibrio* decreased by 1.20%, 0.57%, and 0.94% at 60 d, 120 d, and 150 d, respectively. *Bacillus* and *Microbacterium* decreased by 0.35% and 1.27% at 60 d, respectively. *Thermomonas* decreased by 3.74% at 120 d. The relative abundance of *Streptomyces* increased and then decreased, and GO was less than the CK at 120 d. The relative abundance of *Parvibaculum* showed a trend of decreasing, increasing, and then decreasing. *Nocardia* decreased by 1.3% and 1.1% at 120 d and 150 d, respectively.

The aforementioned dominant bacterial genera have been reported multiple times to participate in the degradation process of organic pollutants. Currently, researchers have isolated a large number of PAH-degrading bacteria from the environment, especially those that degrade high molecular weight PAHs. This includes endophytic bacterial genera such as *Mycobacterium*, *Bacillus*, *Sphingomonas*, *Streptomyces*, *Dokdonella*, *Devosia*, *Flavobacterium*, and *Microbacterium* that we detected in the roots of Fire Phoenix. Most of these dominant genera are known to carry the PAH-RHDα gene and have been demonstrated to be efficient PAH degraders [42,43,44]. These functional bacterial genera have been reported to be involved in the degradation process of PAH components such as naphthalene, phenanthrene, anthracene, fluoranthene, pyrene, benzo[a]anthracene, chrysene, and benzo[a]pyrene and have been confirmed to be related to PAH degradation [43,44,45,46,47,48]. Mafiana et al. explored the relationship between petroleum contamination and soil microbial community structure in three oilfield soils in the Niger Delta and found that the KCM-B-112 bacterial genus is the main dominant genus in the petroleum-contaminated soil microbial community, suggesting that it has good tolerance to petroleum hydrocarbons [45]. *Mycobacterium* is reported to be able to mineralize pyrene and have good degradation effects on high molecular weight PAHs such as phenanthrene, anthracene, and fluoranthene and is often used for the biotreatment of PAHs, petroleum hydrocarbon-contaminated soil, and wastewater [46]. Mahanty et al. reported the degradation capability of *Mycobacterium* on complex PAH pollution of anthracene, naphthalene, and pyrene, with removal rates of 54–81% for low concentration PAHs and 67–89% for high concentration PAHs [47]. *Bacillus* played an important role in the degradation process of PAHs, mainly relying on its strong adaptability and resistance to adverse stress. A large number of studies have confirmed that *Bacillus* has great potential for degrading PAHs. Li et al. screened a group of bacteria with the function of degrading PAHs from petroleum-contaminated soil, which included *Bacillus*, *Sphingomonas*, and *Pseudomonas*, and found that they were able to degrade 100% of the fluorene in 3 d, 98.93% of phenanthrene in 7 d, and 98.93% of phenanthrene in 9 d [48]. *Bacillus* can also degrade persistent organic pollutants such as crude oil-contaminated hydrocarbons and PAHs, which can be used for the remediation of petroleum-contaminated sites. Additionally, *Bacillus* can grow and reproduce rapidly under PAH-contaminated conditions and has good removal effects on pyrene, phenanthrene, fluoranthene, anthracene, and benzo[a]pyrene [49]. Studies have revealed that the bacterium *Sphingomonas* exhibits remarkable proficiency in degrading polycyclic aromatic hydrocarbons (PAHs). After a 16-h incubation period with PAHs at a starting concentration of 10 mg·L^−1^, *Sphingomonas* achieved degradation rates of 80% for pyrene, 72.9% for benz[a]anthracene, 31.5% for chrysene, 33.3% for benzo[a]pyrene, 12.5% for benzo[b]fluoranthene, and 7.8% for dibenz[a,h]anthracene [50]. Moreover, this bacterium is capable of using naphthalene, phenanthrene, anthracene, and fluoranthene as exclusive carbon sources, and it can co-metabolize other PAHs such as fluorene, pyrene, benz[a]anthracene, chrysene, and benzo[a]pyrene, showcasing its potential for bioremediation applications [51]. *Streptomyces* has a strong ability to degrade PAHs. Balachandran et al. isolated *Streptomyces* from soil contaminated with PAHs in a certain area of India, showing that *Streptomyces* can degrade petroleum and PAHs. After incubation for 7 d, the degradation rates of naphthalene, diesel, and phenanthrene reached 98.25%, 99.14%, and 17.5%, respectively [2]. In summary, these endophytic bacteria with degradation capabilities participate in the degradation of PAHs in conjunction with the induction of GO.

### 2.4. Community LEfSe Analysis

**Figure 3:**
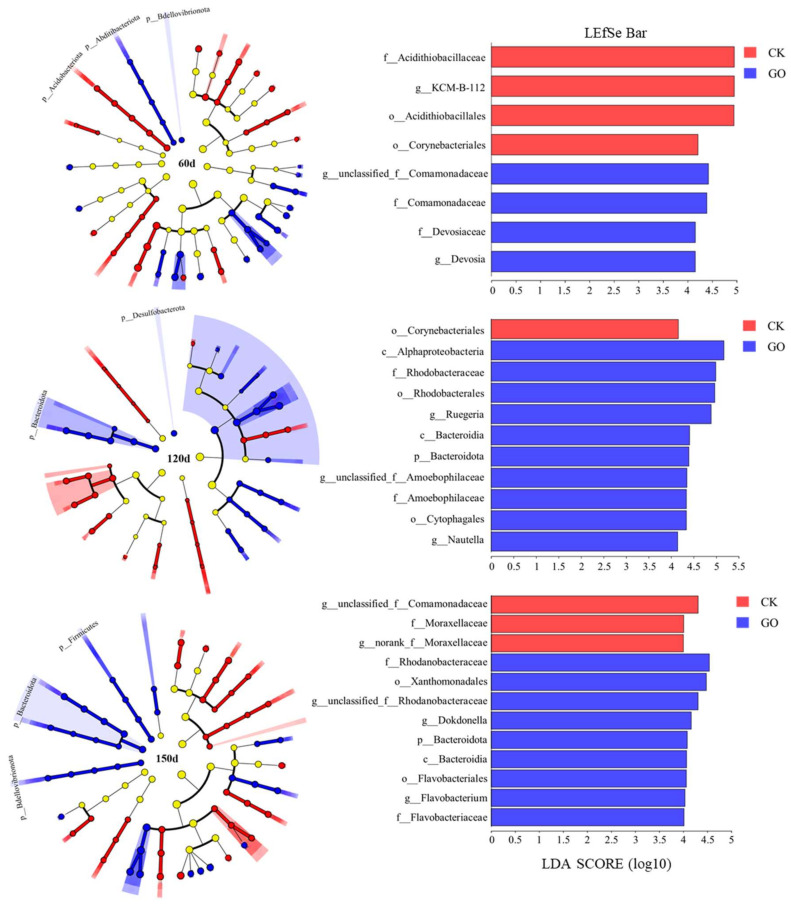
LEfSe analysis of the endophytic bacterial community in the CK and GO samples. The analysis is conducted with linear discriminant analysis (LDA) values set to exceed 4. The nonparametric Kruskal–Wallis rank-sum test is used to discern significant differences. The species level from phylum to genus is displayed in the legend. In the branching graph, each node represents a distinct taxon. Taxa with significant differences within the sample group are denoted by circles colored to match the respective group, while taxa without significant differences are indicated by yellow circles.

The LEfSe method enables the analysis of species composition differences between groups, pinpointing distinct microbial species. By employing the LEfSe approach for biomarker analysis, we assessed the specific endophytic bacteria ranging from phylum to genus within the CK and GO samples. The primary objective was to identify biomarkers present in the root samples of our investigation. The results for each group were illustrated in cladograms, and the significant biomarkers were highlighted with an LDA score threshold of four or higher, as depicted in Figure 3. There were eight groups of endophytic bacteria at 60 d, 11 groups at 120 d, and 12 groups at 150 d which showed statistically significant differences across various branches (LDA > 4.0, *p* < 0.05). At the 60-d mark, GO treatment was associated with a notable enrichment in Comamonadaceae and Devosiaceae at the family level, and at the genus level, unclassified Comamonadaceae and *Devosia,* when compared to the CK. By day 120, the CK samples were characterized by a significant presence of Corynebacteriales at the order level, whereas GO samples displayed enrichment across 10 endophytic bacterial groups, including Bacteroidota at the phylum level, Alphaproteobacteria and Bacteroidia at the class level, Rhodobacterales and Cytophagales at the order level, Rhodobacteraceae and Amoebophilaceae at the family level, and *Ruegeria*, unclassified Amoebophilaceae, and *Nautella* at the genus level. At 150 d, only three groups of endophytic bacteria were enriched in the CK, in contrast to nine groups which were enriched in the GOG. The most significant contributors in the GOG were Rhodanobacteraceae at the family level and Xanthomonadales at the order level, followed by unclassified Rhodanobacteraceae, *Dokdonella*, and *Flavobacterium* at the genus level. Additionally, Bacteroidota, Bacteroidia, Flavobacteriales, and Flavobacteriaceae were also significantly enriched. Overall, the addition of GO resulted in significant differences in the composition of endophytic bacteria, reflecting to some extent the impact of GO on the plant endophytic bacterial community under PAH contamination. Moreover, most of the identified endophytic bacteria with differential abundance in the samples possess the ability to degrade PAHs, which may be the reason for the enhanced degradation of polycyclic aromatic hydrocarbons induced by GO.

### 2.5. PICRUSt2 Functional Prediction Analysis

**Figure 4:**
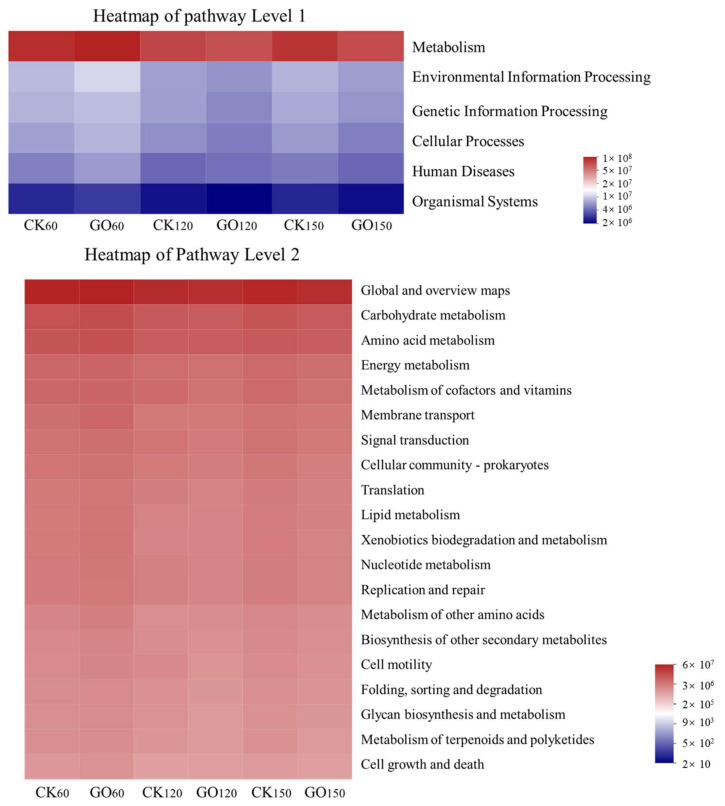
Heatmap of PICRUSt2-based predicted functions for the CK and GO.

PICRUSt2 (http://huttenhower.sph.harvard.edu/galaxy, accessed on 22 April 2024) is a bioinformatic tool designed to forecast the functional profiles of microbial communities, offering insights into their collective functional potential. The potential functions of the endophytic bacterial community in Fire Phoenix roots were predicted using PICRUSt2. As shown in Figure 4, based on KEGG orthology analysis, the endophytic bacterial community primarily exhibits some functions with high relative abundance at the first hierarchical level, with each predicted function showing similar relative abundance between the CK and GO groups. In descending order of relative abundance, they are as follows: Metabolism (75.86~76.73%), Environmental Information Processing (6.25~6.91%), Genetic Information Processing (5.68~6.17%), Cellular Processes (5.11~5.48%), and Organismal Systems (1.91~2.04%). The top three predicted functions account for over 88.34% of the relative abundance. Analysis of the endophytic bacterial community functions over different periods revealed no significant differences in the abundance of these six first-level predicted genes (*p* > 0.05). Further analysis of the predicted genes at the secondary functional level showed that the top 10 functions in terms of abundance at the second level are as follows: Global and Overview Maps (39.61~40.37%), Carbohydrate Metabolism (8.64~9.39%), Amino Acid Metabolism (7.80~8.26%), Energy Metabolism (4.33~4.50%), Metabolism of Cofactors and Vitamins (4.12~4.31%), Membrane Transport (2.65~2.91%), Signal Transduction (2.45~2.79%), Cellular Community Prokaryotes (2.45~2.79%), Translation (2.62~2.68%), and Lipid Metabolism (2.29~2.47%). Among these, six metabolic functions, namely Global and Overview Maps, Carbohydrate Metabolism, Amino Acid Metabolism, Energy Metabolism, Metabolism of Cofactors and Vitamins, and Lipid Metabolism, account for 59.91~69.04% of the total functional information. Notably, functions such as Carbohydrate Metabolism, Amino Acid Metabolism, Energy Metabolism, and Lipid Metabolism are more abundant in the GOG. These metabolic activities primarily serve to decompose pollutants into carbon and nitrogen sources, among other energy substances, further indicating significant changes in the metabolism-related functions of endophytic bacteria guided by GO. Therefore, GO induces the endophytic bacterial community to carry out many active metabolic activities related to metabolic functions during the degradation of PAHs. This study provides a preliminary analysis of the functional profile of the endophytic bacterial community based on PICRUSt2. Considering the limitations of PICRUSt2, future research should integrate metagenomic sequencing techniques to further investigate the functions of the endophytic bacterial community during the PAH degradation process.

### 2.6. Correlation Analysis

**Figure 5:**
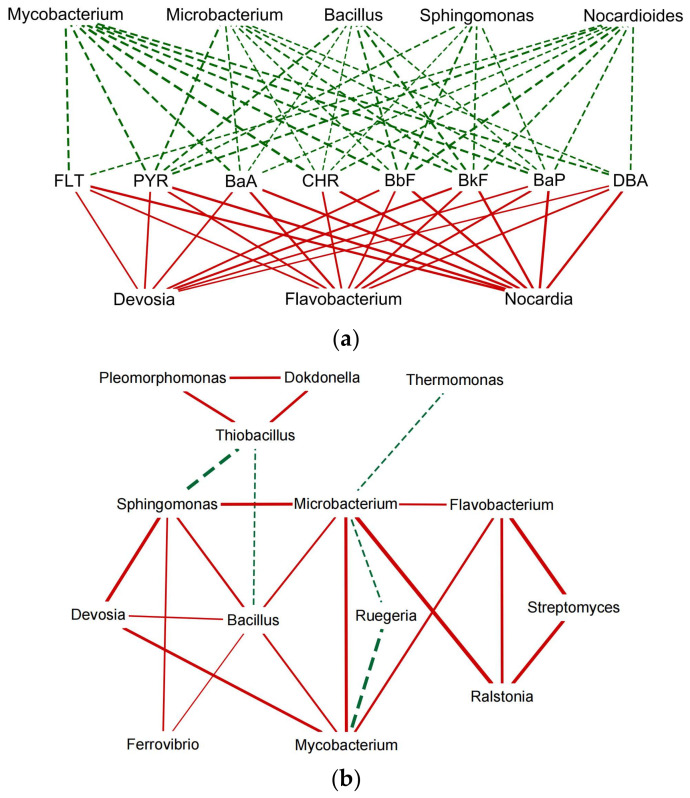
(**a**) Network diagram of the correlation between PAH degradation rates and endophytic bacteria at the genus level. (**b**) Correlation diagram of the endophytic bacterial symbiotic relationship network at the genus level. Red solid lines represent positive correlations, green dashed lines represent negative correlations, and the thickness of the lines represents the size of the correlation.

Based on the correlation analysis of the Cytoscape network at the genus level (Figure 5a), it was found that certain endophytic bacteria genera were significantly correlated with the removal rate of PAHs (including FLT, PYR, BaA, CHR, BbF, BkF, BaP, and DBA) (*p* < 0.05). The endophytic bacteria that had a significant positive correlation with the removal rate of PAHs were *Devosia* (It came from Proteobacteria) (rFLT = 0.58, rPYR = 0.64, rBaA = 0.63, rBaP = 0.60, rDBA = 0.55), *Flavobacterium* (It came from Bacteroidota) (rFLT = 0.62, rPYR = 0.67, rBaA = 0.74, rCHR = 0.66, rBbF = 0.64, rBkF = 0.71, rBaP = 0.69, rDBA = 0.64), and *Nocardia* (It came from Actinobacteria) (rFLT = 0.76, rPYR = 0.78, rBaA = 0.76, rCHR = 0.77, rBbF = 0.79, rBkF = 0.77, rBaP = 0.80, rDBA = 0.77) (*p* < 0.05). It has been reported that *Devosia*, *Flavobacterium*, and *Nocardia* can degrade high-ring PAHs as carbon and energy sources [40]. The endophytic bacteria that showed a significant negative correlation with the removal rate of PAHs were *Mycobacterium* (rPYR = −0.71, rBaA = −0.71, rCHR = −0.80, rBbF = −0.75, rBkF = −0.70, rBaP = −0.72, rDBA = −0.67), *Microbacterium* (rPYR = −0.64, rBaA = −0.66, rCHR = −0.65, rBbF = −0.72, rBkF = −0.66, rBaP = −0.66, rDBA = −0.63), and *Nocardioides* (rPYR = −0.69, rBaA = −0.56, rCHR = −0.61, rBbF = −0.75, rBkF = −0.63, rBaP = −0.61, rDBA = −0.60), they were from Actinobacteria. There were also *Sphingomonas* (rPYR = −0.48, rCHR = −0.38, rBbF = −0.65, rBkF = −0.59, rBaP = −0.56) (It came from Proteobacteria) and *Bacillus* (rPYR = −0.67, rCHR = −0.56, rBbF = −0.67, rBkF = −0.72, rBaP = −0.65) (It came from Firmicutes). These endophytic bacteria have been reported to degrade high-ring PAHs by co-metabolizing with other organisms [40].

In Figure 5b, correlation analysis between endophytic bacteria at the genus level reveals the interactions between specific bacterial genera capable of degrading PAHs. The results showed significant positive correlations between *Mycobacterium, Microbacterium, Flavobacterium, Sphingomonas, Devosia, Bacillus, Streptomyces,* and *Ralstonia* under the influence of GO. This suggests that these endophytic bacteria are able to coexist in the roots and, induced by GO, they not only participate in the degradation process of PAHs in the root system, but also interact and respond to each other to form a close consortium that jointly promotes the degradation of PAHs. The synergistic interactions between these bacteria may be achieved through a series of complex biochemical pathways and signaling mechanisms, where some of them may provide essential enzymes or metabolites that others may utilize for further degradation of PAHs. For example, *Flavobacterium* may secrete specific enzymes to break down larger PAH molecules, whereas *Sphingomonas* may utilize these breakdown products as energy sources [48,50]. In addition, bacteria like *Bacillus* and *Streptomyces* may provide stability and support under harmful environmental conditions due to their strong adaptive capacity and resistance [49,50]. Ultimately, the interactions and synergistic effects of this microbial community not only enhance the rate of PAH degradation but may also improve the overall health and biodiversity of the roots. The collective action of these endophytic bacteria, especially when guided by GO, served as a key regulator of Fire Phoenix in PAH pollution remediation, demonstrating the great potential of plant–microbe interactions in environmental remediation.

## 3. Materials and Methods

### 3.1. Plant and Soil

Test plant: Fire Phoenix, originating from the USA, belongs to the biological *Festuca* spp. and is a mixed genus of fescue hybridized by three dominant species of *Festuca arundinacea* Schreb., *Festuca elata* Keng ex E. Alexeev, and *Festuca gigantea* (L.) Vill. It has a well-developed and robust root system, strong adaptability, extends deeper into the soil, and is a widely distributed perennial graminaceous plant with the advantages of cold hardiness, drought resistance, and strong resistance to adversity, as well as displaying good remediation effects on petroleum-based contaminated soils. Fire Phoenix seeds were purchased from Beijing Crowe Seed Co. (Beijing, China) with 98% purity and over 85% germination rate.

Test soil: The PAH-contaminated soil used in this study was obtained from the abandoned and aged petroleum hydrocarbon-contaminated soil of the First Oil Extraction Plant of Dagang Oilfield (38◦75′ N, 117◦58′ E) in Tianjin City, China. The total concentration range of the eight PAHs identified as priority pollutants by the U.S. Environmental Protection Agency (EPA) was 160.35~225.68 mg·kg^−1^ in this PAH-contaminated soil, which contained four 4-ring PAHs, namely, Fluoranthene (FLT), Pyrene (PYR), Benzo[a]anthracene (BaA), and Chrysene (CHR), and four 5-ring PAHs, Benzo[b]fluoranthene (BbF), Benzo[k]fluoranthene (BkF), Benzo[a]pyrene (BaP), and Dibenzo[a,h]anthracene (DBA). Due to the sticky soil and high salinity of this crude oil-contaminated soil, it is not suitable for the healthy growth and survival of plants when planted directly. Therefore, by adding non-polluted clean soil to the original polluted soil, the concentration of pollutants was diluted to the polluted range suitable for plant growth. The clean soil was collected from the surface soil (soil sampling depth 0–20 cm) in the northern area of Fushun City, China (41°78′ N, 123°47′ E). According to the results of the field investigation, which showed that there was no pollution history in the area, PAHs were not detected in the collected soil. Based on the growth of plants in the pre-experiment, the original contaminated soil was diluted to a PAH contamination concentration of 100 ± 10.45 mg·kg^−1^, and the contaminated soil was aged for 1 month prior to the experiment. The basic physical and chemical properties of the experimental soil were as follows: pH 6.70; organic matter content 19.8 g·kg^−1^; total carbon 12.8 g·kg^−1^; total nitrogen 0.92 g·kg^−1^; effective phosphorus 11.0 mg·kg^−1^; effective potassium 54.35 mg·kg^−1^; and cation exchange 18.56 cmol·kg^−1^.

### 3.2. Graphene Oxide

The nanomaterial used in this experiment was multilayer graphene oxide, which was purchased from Suzhou Tanfeng Graphene Technology Co., Ltd. (Suzhou, China). Its appearance was black-brown powder, purity > 95%, and the composition was C content 68.44%, O content 30.92%, and S content 0.63%. Its thickness was 3.4–7 nm, the diameter of the sheet was 10–50 μm, the number of layers was 6–10, and the specific surface area was 100–300 m^2^·g^−1^.

### 3.3. Experimental Design

The pot experiment was conducted at the National Field Scientific Observation and Research Station of Shenyang Farmland Ecosystem, China (41°31′ N, 123°24′ E). Two treatments, control (CK) and GO (0.4 g·kg^−1^), were established in this experiment. After addition of graphene oxide, plant and soil samples were analyzed on day 60, 120, and 150, respectively. The experimental configurations and planting methods used refer to Li et al. [40]. Pure watering was used to maintain soil moisture at 60% of the field holding capacity (*w*:*w*). The light duration was 14 ± 2 h per day, the daily temperature was about 28 ± 4 °C, the night temperature was about 19 ± 2 °C, and the photosynthesized photon flux density was about 400–500 μmol·m^−2^·s^−1^. Each treatment had 6 replicates.

### 3.4. PAHs Extraction and Analysis

PAHs were extracted from plant roots with reference to the method of Li et al. [40]. The concentrations of the eight target PAHs were determined using a gas chromatograph mass spectrometer (GC-MS, Agilent 6890N, Santa Clara, CA, USA). An in-depth description of the specific steps and methodology is provided in Supplementary Materials. Quantification of PAHs in 8 was performed by comparison with an established 6-point standard curve. PAHs in soil were extracted and measured according to the same procedures and methods as for plant roots.

### 3.5. Community Structure and Diversity Analysis of Endophytic Bacteria

The root samples of Fire Phoenix were preserved in liquid nitrogen to maintain their frozen state until the DNA extraction process. The extraction of plant root DNA was facilitated by the FastDNA^®^ Spin Kit (MPBIO, Santa Ana, CA, USA) for Soil. The extracted DNA’s integrity was confirmed via 1% agarose gel electrophoresis, and its concentration and purity were assessed using a NanoDrop 2000 UV–vis spectrophotometer (Thermo Scientific, Wilmington, DE, USA). Amplification of the endophytic bacterial 16S rRNA gene was achieved with the primer pair 799F (5′-AACMGGATTAGATACCCKG-3′) and 1193 R (5′-ACGTCATCCCCACCTTCC-3′) targeting the V5-V6-V7 region [52]. High-throughput sequencing of the purified amplicons was performed by Shanghai Majorbio Bio-pharm Technology Co., Ltd. (Shanghai, China) using an Illumina MiSeq platform (San Diego, CA, USA). Additionally, sequencing data was deposited in the National Center for Biotechnology Information under accession number PRJNA1108426.

### 3.6. Statistical Analysis

All data analyses were repeated three times, with statistical significance established at the *p* < 0.05 level. Data processing and analysis were performed using SPSS 20.0 and Origin 9.0. We utilized one-way ANOVA, preceded by tests for normality (Shapiro–Wilk) and homogeneity of variances (Levene’s test). Post hoc comparisons were performed using the Least Significant Difference (LSD) test with a significance threshold of *p* < 0.05. For alpha diversity analysis, we utilized the Mothur v.1.30.1 software package and the QIIME pipeline (QIIME2 v2022.2). To assess differences in index values across multiple groups, we applied Analysis of Variance (ANOVA) followed by a post-hoc Tukey test. Visualizations included the following: community distribution bar maps for each sample, based on taxonomic data, created using the R programming language; Venn diagrams at the OTU level, also generated using R; a community heat map produced using the vegan package in R; a visual circle depicting the correspondence between samples and species, created using Circos-0.67-7 software; Linear Discriminant Analysis Effect Size (LEfSe) analysis results presented in visual graphs; and correlation network plots generated using Cytoscape 3.7.2.

## 4. Conclusions

This study verified the effectiveness of GO in enhancing the phytoremediation of PAH-contaminated soil. GO significantly improved the degradation of PAHs and enhanced the uptake capacity of plants. The addition of GO also optimized the community of endophytic bacteria within the roots. Compared to the control, the GO group exhibited higher bacterial diversity indices, such as Shannon and Simpson indices, at multiple time points, indicating that GO enriched the endophytic bacterial community and significantly increased the relative abundance of specific bacterial genera, especially those capable of degrading PAHs. Genera such as *Devosia*, *Flavobacterium*, and *Nocardia* showed a significant positive correlation with the removal rate of PAHs, playing a pivotal role in the degradation process. LEfSe analysis and correlation studies further revealed the influence of GO on specific endophytic bacteria, such as *Mycobacterium*, *Microbacterium*, *Flavobacterium*, *Sphingomonas*, *Devosia*, *Bacillus*, and *Streptomyces*, which coexist and interact under the influence of GO, synergistically degrading PAHs. These bacteria may serve as key biological markers in the degradation process of PAHs. These findings underscore the significant role of GO in plant–microbe interactions, offering new strategies for the biodegradation of PAH-contaminated soils. Future research may explore how to optimize the application of GO to further enhance the synergistic degradation efficiency of these endophytic bacteria, providing more effective strategies for the bioremediation of PAH-contaminated soils.

## Figures and Tables

**Table 1:** Endophytic bacterial diversity indices of the CK and GO at different time periods.

Time	Sample	Sequences	OTU	Shannon	Simpson	Ace	Chao
60 d	CK	42,386	1015	3.9599	0.0304	294.86	305.86
	GO	45,759	1119	4.8004	0.0259	397.66	398.03
120 d	CK	35,119	1326	2.9597	0.0838	422.79	444.08
	GO	39,999	1468	3.7685	0.0605	447.00	465.99
150 d	CK	33,227	1423	4.0175	0.0622	443.91	409.85
	GO	36,008	1542	4.2140	0.0504	464.77	463.12

## Data Availability

Data are contained within the article.

## Supplementary Materials

The following supporting information can be downloaded at: https://www.mdpi.com/article/10.3390/molecules29102342/s1, Table S1: Initial content of individual PAHs in the soil.
