# Supplementary material for: Effects of Graphene Oxide on Endophytic Bacteria Population Characteristics in Plants from Soils Contaminated by Polycyclic Aromatic Hydrocarbons
Source: Molecules. 2024 May 16;29(10):2342. doi: 10.3390/molecules29102342 (PMC11123924; doi:10.3390/molecules29102342)
Supplement: Supplementary file 1 [file molecules-29-02342-s001.zip › molecules-3000406-supplementary.pdf]

Effects of Graphene Oxide on Endophytic Bacteria Population Characteristics in Plants from Soils Contaminated by Polycyclic Aromatic Hydrocarbons

Xingxing Zhou [1,†], Bo Zhang [2,†], Qingzhu Meng [3] and Lingmei Li [4,*]

[1] College of Architecture and Environment, Ningxia Institute of Science and Technology, Shizuishan 753000, China; 2010419@stu.neu.edu.cn
[2] Key Laboratory of Ministry of Education on Safe Mining of Deep Metal Mines, Northeastern University, Shenyang 110819, China; 2010420@stu.neu.edu.cn
[3] College of Material Science and Green Technologies, Kazakh-British Technical University, Almaty 050000, Kazakhstan; q_meng@kbtu.kz
[4] College of Life Science, Shenyang Normal University, Shenyang 110034, China
* Correspondence: lilingmei0809@163.com or lilingmei17@mails.ucas.ac.cn

† These authors contributed equally to this work.

Table S1. Initial content of individual PAHs in the soil.

Compounds	Content ($mg \cdot kg^{-1}$)
FLT	8.47780
PYR	10.2621
BaA	7.83520
CHR	11.6332
BbF	5.58360
BkF	23.8948
BaP	20.7803
DBA	11.5427
Total content ($mg \cdot kg^{-1}$)	100.0097

PAHs extraction and analysis

PAHs were extracted from plant roots with reference to the method of Li et al [1]. The concentrations of the eight target PAHs were determined using a gas chromatograph mass spectrometer (GC-MS, Agilent 6890N, USA). PAHs were extracted from plant roots using Soxhlet extraction method by weighing 0.5 g of dried plant roots into a 25 ml centrifuge tube and extracting them with 10 ml of methanol solution in an ultrasonic bath for 30 min, with the water temperature kept below 40°C, for three consecutive times. The extract was centrifuged at 4000 rpm for 5 min to separate the supernatant from the plant root system. The supernatant was separated and purified by a 1:2 silica gel/alumina (4 g/8 g) chromatographic column, and the alkanes were washed off with hexane, then eluted twice consecutively with a mixture of 50 mL of hexane and dichloromethane (v/v=1:1), and the eluate was collected, rotary evaporated, and then the extracts were fixed and concentrated with chromatographically pure hexane for PAHs analysis.

A gas chromatography mass spectrometer (GC-MS, Agilent 6890N, USA) equipped with an HP-5975B mass-selective detector and a DB-5 capillary column (60 m × 0.18 mm I.D. × 0.25 μm film thickness) was used to analyze the final concentrated extracts from the root samples of P. firnum in the selected ion monitoring mode. The GC heating procedure was as follows: an initial oven temperature of 80°C (hold for 2 min), ramping up to 255°C at 15°C·min^{-1}, holding for 1 min, then ramping up to 265°C at 1°C·min^{-1}, holding for 1 min, and finally ramping up to 295°C at 2.5°C·min^{-1}, holding for 3 min; the inlet temperature was 290°C and the detector temperature was 325°C. Helium was used as the carrier gas with a gas flow rate of 0.8 mL·min^{-1}, and the specimen was injected in an unshunted manner into 1.0 μL. The mass spectrometer was set up in full-scan mode with a scanning range of m/z from 35 to 480. On the basis of the full-scan, two to three options for qualitative and quantitative analyses were selected for the elution time (selected ions) of each test component. Quantification of PAHs in 8 was performed by comparison with an established 6-point standard curve. PAHs in soil were extracted and measured according to the same procedures and methods as for plant roots.
